# Supplementary material for: The experience of a tele-operated avatar being touched increases operator’s sense of discomfort
Source: PeerJ Comput Sci. 2024 Mar 19;10:e1926. doi: 10.7717/peerj-cs.1926 (PMC11042036; doi:10.7717/peerj-cs.1926)
Supplement: Supplemental Information 2 [file peerj-cs-10-1926-s002.pdf]

アンケート

Google にログインすると作業内容を保存できます。詳細

* 必須の質問です

氏名 *

回答を入力

以下の設問にお答えください.
1：全くそう思わない
2：あまりそう思わない
3：ややそう思わない
4：どちらともいえない
5：ややそう思う
6：かなりそう思う
7：とてもそう思う

[Q1] アバターの身体が触れられたときあたかも自分の身体が触れられたように感じた *

	1	2	3	4	5	6	7	
全くそう思わない	○	○	○	○	○	○	○	とてもそう思う

[Q2] アバターの身体が触れられたとき不快感があった *

	1	2	3	4	5	6	7	
全くそう思わない	○	○	○	○	○	○	○	とてもそう思う

[Q3] アバターの身体をあたかも自分の身体のように感じた *

	1	2	3	4	5	6	7	
全くそう思わない	○	○	○	○	○	○	○	とてもそう思う

[Q4] アバターは自分の意思に従って動いているように感じた *

	1	2	3	4	5	6	7	
全くそう思わない	○	○	○	○	○	○	○	とてもそう思う

[Q5] もし同じ状況で遠隔操作システムを利用する機会があれば、近いうちにまたこの遠隔操作システムを利用すると思う。 *

	1	2	3	4	5	6	7	
全くそう思わない	○	○	○	○	○	○	○	とてもそう思う

[Q6] もし同じ状況で遠隔操作システムを利用する機会があれば、数日中に間違いなくこの遠隔操作システムを利用する。 *

	1	2	3	4	5	6	7	
全くそう思わない	○	○	○	○	○	○	○	とてもそう思う

[Q7] もし同じ状況で遠隔操作システムを利用する機会があれば、この遠隔操作システムを利用する計画を立ててみたい。 *

	1	2	3	4	5	6	7	
全くそう思わない	○	○	○	○	○	○	○	とてもそう思う

[Q8] 集中して説明を行えた *

	1	2	3	4	5	6	7	
全くそう思わない	○	○	○	○	○	○	○	とてもそう思う

[Q9] 自分のアバターが触られている様子が分かりやすかった *

	1	2	3	4	5	6	7	
全くそう思わない	○	○	○	○	○	○	○	とてもそう思う

以下のスケールに基づいてこのアバターの印象を評価してください。

[L1] *

	1	2	3	4	5	
嫌い	○	○	○	○	○	好き

[L2] *

	1	2	3	4	5	
親しみにくい	○	○	○	○	○	親しみやすい

[L3] *

	1	2	3	4	5	
不親切な	○	○	○	○	○	親切な

[L4] *

	1	2	3	4	5	
不愉快な	○	○	○	○	○	愉快な

[L5] *

	1	2	3	4	5	
ひどい	○	○	○	○	○	良い

送信　　フォームをクリア

Google フォームでパスワードを送信しないでください。

このコンテンツは Google が作成または承認したものではありません。不正行為の報告 - 利用規約 - プライバシーポリシー

Google フォーム

Questionnaire

Sign in to Google to save your progress. Learn more

* Indicates required question

Name *

Your answer

Please answer the following questions.
1: Strongly disagree
2: Disagree
3: Somewhat disagree
4: Neither agree or disagree
5: Somewhat agree
6: Agree
7: Strongly agree

[Q1]
I felt as if my own body was being touched when the avatar's body was touched. *

	1	2	3	4	5	6	7	
Strongly disagree	○	○	○	○	○	○	○	Strongly agree

[Q2] I felt discomfort when the avatar's body was touched. *

	1	2	3	4	5	6	7	
Strongly disagree	○	○	○	○	○	○	○	Strongly agree

[Q3] I felt as if the avatar's body was my own body. *

	1	2	3	4	5	6	7	
Strongly disagree	○	○	○	○	○	○	○	Strongly agree

[Q4] I felt as if the avatar moved as if obeying my will. *

	1	2	3	4	5	6	7	
Strongly disagree	○	○	○	○	○	○	○	Strongly agree

[Q5] I think I'll use the tele-operated system next few days *

	1	2	3	4	5	6	7	
Strongly disagree	○	○	○	○	○	○	○	Strongly agree

[Q6] I am certain to use the tele-operated system the next few days *

	1	2	3	4	5	6	7	
Strongly disagree	○	○	○	○	○	○	○	Strongly agree

[Q7] I'm planning to use the tele-operated system the next few days *

	1	2	3	4	5	6	7	
Strongly disagree	○	○	○	○	○	○	○	Strongly agree

[Q8] I was able to maintain my concentration while I was explaining. *

	1	2	3	4	5	6	7	
Strongly disagree	○	○	○	○	○	○	○	Strongly agree

[Q9] It was easy to sense when my avatar was being touched. *

	1	2	3	4	5	6	7	
Strongly disagree	○	○	○	○	○	○	○	Strongly agree

Please rate your impression of the avatar on these scales:

[L1] *

	1	2	3	4	5	
Dislike	○	○	○	○	○	Like

[L2] *

	1	2	3	4	5	
Unfriendly	○	○	○	○	○	Friendly

[L3] *

	1	2	3	4	5	
Unkind	○	○	○	○	○	Kind

[L4] *

	1	2	3	4	5	
Unpleasant	○	○	○	○	○	Pleasant

[L5] *

	1	2	3	4	5	
Awful	○	○	○	○	○	Nice

Submit   Clear form

Never submit passwords through Google Forms.

This content is neither created nor endorsed by Google. Report Abuse - Terms of Service - Privacy Policy

Google Forms
